# Volume loss during muscle reinnervation surgery is correlated with reduced CMAP amplitude but not reduced force output in a rat hindlimb model

**DOI:** 10.3389/fphys.2024.1328520

**Published:** 2024-02-15

**Authors:** Alexis L. Lowe, Maria V. Rivera Santana, Taylor Bopp, Kiara N. Quinn, Johnnie Johnson, Christopher Ward, Tae Hwan Chung, Sami Tuffaha, Nitish V. Thakor

**Affiliations:** ^1^ Department of Biomedical Engineering, Johns Hopkins School of Medicine, Baltimore, MD, United States; ^2^ Department of Biology, University of Puerto Rico at Mayagüez, Mayagüez, PR, United States; ^3^ Department of Physical Medicine and Rehabilitation, Johns Hopkins School of Medicine, Baltimore, MD, United States; ^4^ Department of Orthopedics, University of Maryland School of Medicine, Baltimore, MD, United States; ^5^ Department of Plastic and Reconstructive Surgery, Johns Hopkins School of Medicine, Baltimore, MD, United States

**Keywords:** muscle reinnervation, isometric tetanic force testing, peripheral nerve, compound motor action potential amplitude, muscle histology

## Abstract

**Introduction:** Muscle reinnervation (MR) surgery offers rehabilitative benefits to amputees by taking severely damaged nerves and providing them with new denervated muscle targets (DMTs). However, the influence of physical changes to muscle tissue during MR surgery on long-term functional outcomes remains understudied.

**Methods:** Our rat hindlimb model of MR surgery utilizes vascularized, directly neurotized DMTs made from the lateral gastrocnemius (LG), which we employed to assess the impact of muscle tissue size on reinnervation outcomes, specifically pairing the DMT with the transected peroneal nerve. We conducted MR surgery with both DMTs at full volume and DMTs with partial volume loss of 500 mg at the time of surgery (*n* = 6 per group) and measured functional outcomes after 100 days of reinnervation. Compound motor action potentials (CMAPs) and isometric tetanic force production was recorded from reinnervated DMTs and compared to contralateral naïve LG muscles as positive controls.

**Results:** Reinnervated DMTs consistently exhibited lower mass than positive controls, while DMTs with partial volume loss showed no significant mass reduction compared to full volume DMTs (*p* = 0.872). CMAP amplitudes were lower on average in reinnervated DMTs, but a broad linear correlation also exists between muscle mass and maximum CMAP amplitude irrespective of surgical group (R^2^ = 0.495). Surprisingly, neither MR group, with or without volume loss, demonstrated decreased force compared to positive controls. The average force output of reinnervated DMTs, as a fraction of the contralateral LG’s force output, approached 100% for both MR groups, a notable deviation from the 9.6% (±6.3%) force output observed in our negative control group at 7 days post-surgery. Tissue histology analysis revealed few significant differences except for a marked decrease in average muscle fiber area of reinnervated DMTs with volume loss compared to positive controls (*p* = 0.001).

**Discussion:** The results from our rat model of MR suggests that tissue electrophysiology (CMAPs) and kinesiology (force production) may recover on different time scales, with volumetric muscle loss at the time of MR surgery not significantly reducing functional outcome measurements for the DMTs after 100 days of reinnervation.

## 1 Introduction

Peripheral nerves are the most regenerative part of the human nervous system, possibly because the nature of being in the body’s periphery increases the risk of physical trauma (Johnson et al., 2005). In certain peripheral cases, like amputation, physical trauma to the limb is so severe that the damaged nerve has lost its original target and now has no clear direction in which to regenerate. In this case, the cut nerve ending becomes a neuroma, a bulbous cap that is a mix of neurons and scar tissue (Hsu and Cohen, 2013). Because the nerve still communicates with the brain, these neuromas can be symptomatic, causing extreme neuropathic pain that impedes daily living (Bailey et al., 2009).

**FIGURE 1:**
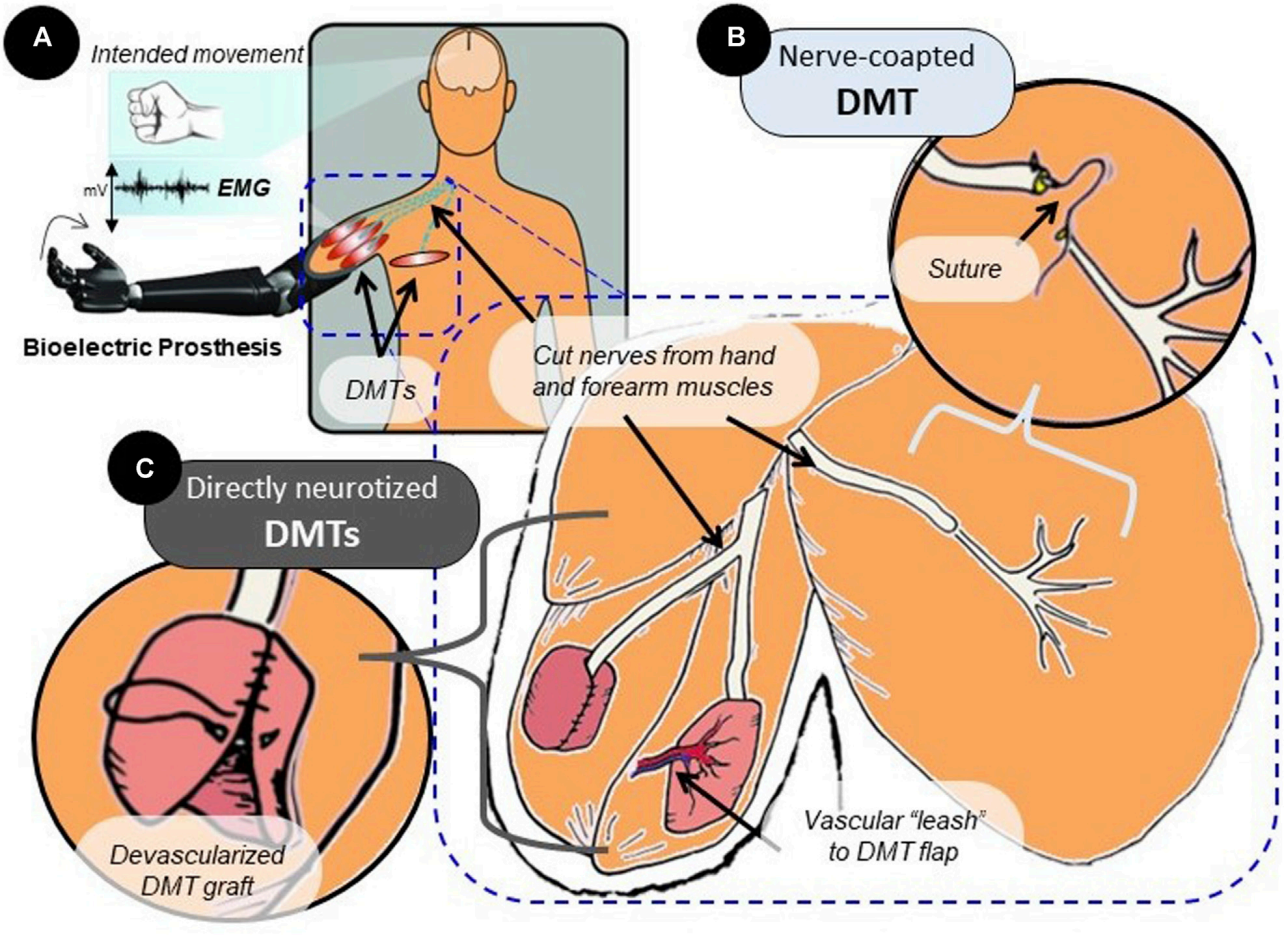
Surgical variations of the MR procedure. **(A)** MR surgery is often performed in amputees to prevent the development of neuropathic pain and amplify motor nerve signals. Over time, the reinnervated muscle will produce EMG signals that reflect activity in the connected neural tissue. **(B)** In surgery, the nerve that has lost its original target post-amputation can be transferred to a nearby area with the available muscle tissue. Suturing the transected nerve end-to-end with the distal nerve stump of a DMT in that area is referred to as nerve coaptation. **(C)** Rather than nerve coaptation, which does not disturb the muscle tissue itself, a free-floating muscle graft taken from another area of the body can be directly wrapped around the cut nerve ending. This is direct nerve-to-muscle neurotization, which represents a different surgical strategy that is used regularly in human subjects. Directly neurotized DMTs are often devascularized, but they can be partially tethered to the vascular system by a “leash” or flap. This enhances tissue survival but reduces spatial freedom for tissue reconstruction.

Surgeons have developed a good treatment for symptomatic neuromas: muscle reinnervation (MR) surgery (Dumanian et al., 2019). In MR, the neuroma is first removed by completely transecting the nerve, and then spare skeletal muscle tissue is re-allocated from elsewhere in the body to give the cut nerve somewhere to grow. This muscle tissue must be relieved of its endogenous innervation and then surgically connected with the new nerve ending. Over the course of months, axons will sprout from the transected nerve and will form new connections with the denervated muscle such that natural electrical impulses sent through the nerve from the brain will stimulate contraction of the newly reinnervated muscle (Kapelner et al., 2016; Dumanian et al., 2019). This is extremely important for developing neuroprosthetic interfaces: devices that can harness the electrical activity of functional tissues (brain, nerve, and muscles) to control an external device, such as a prosthetic limb (Figure 1A) (Cordella et al., 2016; Irwin et al., 2016; Kapelner et al., 2016; Farina et al., 2017; Zbinden et al., 2023). The utility of MR surgery for rehabilitation from amputation is, therefore, two-fold: 1) it reduces neuropathic pain from the affected nerve and 2) it provides a robust signal source, reinnervated muscle, for neuroprosthesis control (Vu et al., 2020).

The creation of denervated muscle targets (DMTs) for the purpose of MR is a process heavily dictated by the surgeon and by the uniqueness of the patient’s own anatomy (Dellon and Aszmann, 2020). The MR procedure was traditionally performed using nearby muscles that were left intact except for their distal motor nerve branch (Figure 1B) (Kuiken et al., 2009). The new donor nerve would be coapted to this distal branch end-to-end, the theory being that the existing endoneurial tube can physically guide the nerve’s growth, and the muscle tissue for the DMT will mostly be undisturbed (Dumanian et al., 2019; Dumanian et al., 2020). This type of end-to-end nerve coaptation can be a physically challenging microsurgery, given the extreme delicacy of nerve tissue and the size-mismatch that is often seen between the donor nerve and recipient nerve being coapted (Valerio et al., 2020; Felder et al., 2022). Because of this pitfall, certain simplified surgical techniques have been developed using direct nerve-to-muscle neurotization (Figure 1C) (Valerio et al., 2020; Scott et al., 2022). For example, skeletal muscle tissue can be surgically removed from anywhere in the body to create a free-floating graft. This small, devascularized graft can be used as a DMT by wrapping it directly around the cut nerve ending (Kubiak et al., 2018; Vu et al., 2020). This technique, clinically referred to as “regenerative peripheral nerve interfaces,” has been indicated through meta-analysis to statistically provide the same neuropathic pain relief as nerve-coapted DMTs (Balcin et al., 2009; Poppler et al., 2018). Similar procedures have been reported in the literature that wrap the DMT around the nerve ending while leaving some muscle tissue or a vascular “leash” intact to provide consistent blood flow to the denervated muscle (Tuffaha et al., 2020; Valerio et al., 2020). No two DMTs are created the same, and with so many possible variations, the standardization of the technique for research purposes presents a major challenge. If not all DMTs are created the same, then are they performing at different levels? Furthermore, do certain surgical variations affect post-reinnervation outcomes?

One surgical variation that is necessary to evaluate is the effect of DMT mass on its functional outcomes after reinnervation. This test is easiest to perform in the untethered, devascularized DMT model of MR, where differently sized muscle tissues can be removed, trimmed, and weighed with precision. After some time of reinnervation, the tissues can be re-accessed and measured for mechanical and electrical signals. This exact experiment was performed in the rat hindlimb by Hu et al. (2020), in which graft DMTs of sizes ranging from 150–1,200 mg were wrapped around the cut peroneal nerve. Four months after the reinnervation surgery, the results showed a strongly *negative* correlation between DMT mass and the amplitude of compound motor action potentials (CMAPs) produced by the DMTs. A CMAP is a type of electromyography (EMG) signal that can be recorded while the subject is anesthetized (or immobile). It represents the summed activity of muscle fibers being simultaneously depolarized when the associated motoneurons are electrically stimulated upstream (Watson and Daube, 2009). A larger CMAP amplitude indicates that a larger number of muscle fibers are actively innervated within the muscle (Watson and Daube, 2009; Ahn et al., 2018). The negative relationship between mass and CMAP amplitude seen in the devascularized DMTs was most strongly affected by the necrosis of muscle tissue observed at the center of the largest DMTs (Hu et al., 2020; Leach et al., 2023). It remains somewhat unclear how changing a DMT’s muscle mass would impact CMAPs and force production in a model that preserves the vasculature of the DMT.

It is easy to assume that a larger DMT mass would also have larger signals post-reinnervation, given the evidence of such relationships in normal skeletal muscle (Knowlton and Hines, 1940; Maughan et al., 1983; Hughes et al., 2018). It is difficult to test the effects of DMT mass on the reinnervation of *vascularized* DMTs because the muscle cannot be removed from the body for precise weighing. In order to explore the relationship between size and functional outcomes in a vascularized DMT model, the independent variable for our experiments must instead be muscle volume *loss*. The DMT mass cannot be directly measured, but we can measure how much tissue we *remove* from the DMT. We used a rat hindlimb model of direct neurotization in a vascularized DMT to measure the functional muscle outcomes (CMAP amplitude and tetanic force output) after 100 days of reinnervation, both with and without volume loss prior to MR surgery.

## 2 Methods and materials

### 2.1 Muscle reinnervation surgical model in the rat hindlimb

**FIGURE 2:**
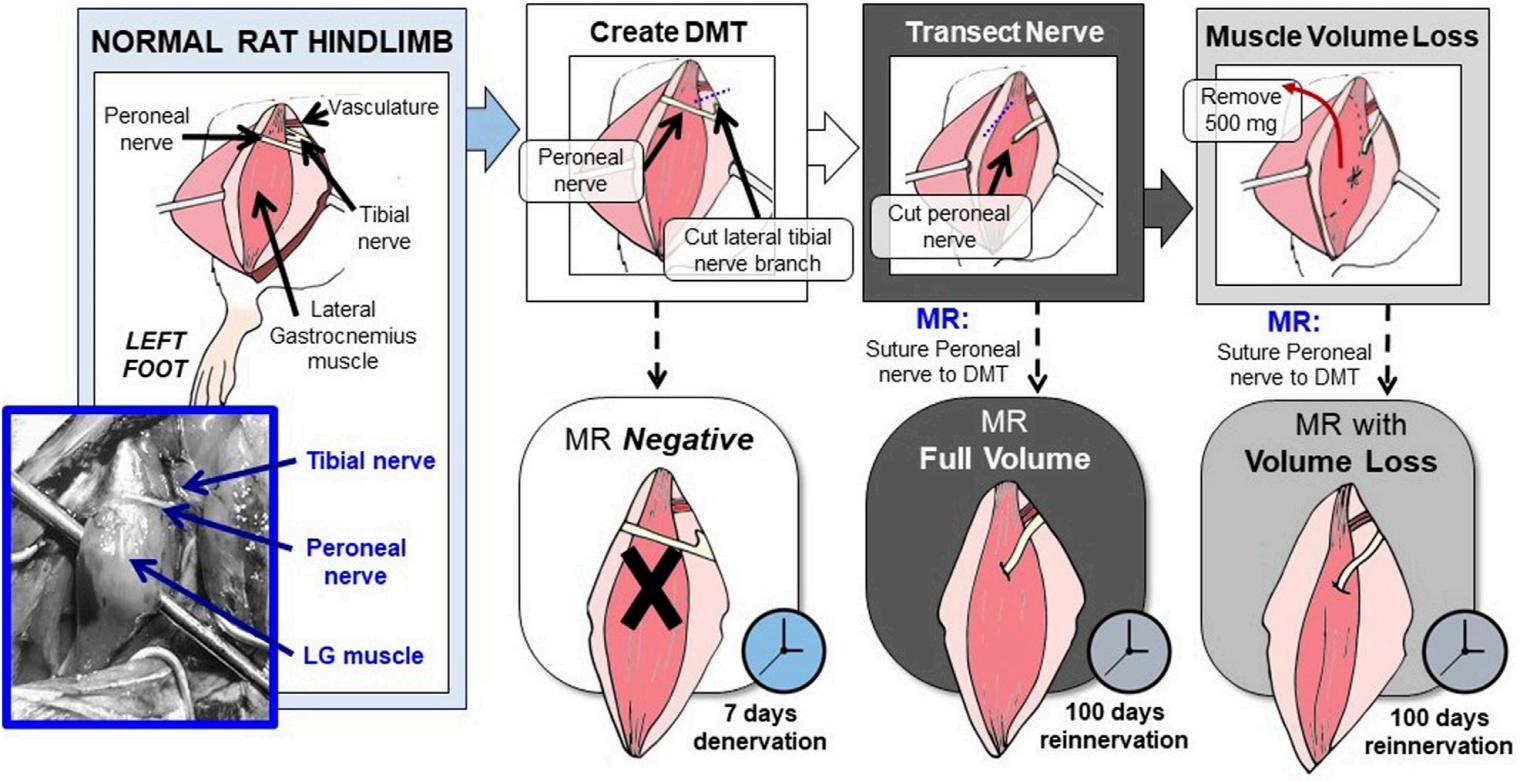
Rat hindlimb model of MR surgery in vascularized, directly neurotized DMTs, with and without volume loss. In this rat hindlimb model of MR, the LG muscle is used as the source for the DMT, and the peroneal nerve acts as the transected nerve. First, the LG must be relieved of its endogenous innervation by cutting the lateral branch of the tibial nerve. This creates the DMT. Then, reinnervation is performed by suturing the cut peroneal nerve directly to the DMT’s epimysium, either with or without volume loss. To model volumetric muscle loss, 500 mg of muscle tissue was removed before neurotization. Both MR groups were given 100 days to reinnervate before functional outcomes were measured. The MR-negative group was given 7 days of denervation time before being measured.

These experiments were performed using 10-week-old Lewis rats, all male. Procedures and compounds were first approved by the Institutional Animal Care and Use Committee. Before surgery, the rats were placed under 3% isoflurane gas anesthesia and then maintained with 2% administered through a nose cone. The rats also received 1.2 mg/kg of buprenorphine SR prior to surgery as an analgesic and then again as needed for treating any post-operative pain. For this experimental model, the left hindlimb’s lateral gastrocnemius (LG) muscle was used as the source for the DMT tissue, and the nearby peroneal nerve acted as the cut nerve (Figure 2). All DMTs were first denervated by transecting the short branch of the tibial nerve, which dives into the lateral gastrocnemius as its endogenous source of motor innervation, first as distally as possible and then again as proximally as possible (without further dividing the sciatic nerve fascicles). This transection denervates the lateral gastrocnemius, as well as the soleus and plantaris, while sparing innervation for the medial gastrocnemius (Badia et al., 2010). The peroneal nerve was transected as distally as possible and carefully retracted. For the *MR-negative* animals (*n* = 6), the peroneal nerve was reflected back and tucked near the sciatic notch, far from contact with the DMT.

For rats with *MR at full volume* (*n* = 6), a small pocket was cut into the DMT tissue’s epimysium, and the end of the peroneal nerve was inserted. The two tissues were sutured at the endoneurium using 9–0 silk sutures. For rats in the *MR with the volume loss* group (*n* = 6), the DMT tissue had to be removed prior to reinnervation. Strips of the muscle tissue were removed longitudinally until a mass of 500 mg had been removed, roughly 1/3 of the tissue. After removing the tissue, MR was performed in the same manner as in the full-volume group. Detailed surgical images can be viewed in Supplementary Figure S1.

### 2.2 Compound motor action potentials

After 100 days of reinnervation (or 7 days of denervation for the MR-negative group), the same rats were placed under isoflurane gas anesthesia, and the left leg DMT tissue was again accessed surgically. The MR-negative group was recorded after 7 days because we observed that after 100 days, the MR-negative groups displayed signs of near-complete reinnervation despite our efforts to provide physical barriers between the DMT and transected nerves. Recording after 7 days allowed for some tissue atrophy and healing to occur without the risk of observing reinnervation. All recordings were performed first on the left leg DMT tissue. This tissue was removed for analysis, and then the same recording procedure was performed on the naïve gastrocnemius muscle of the same rat’s right leg, acting as a positive control.

**FIGURE 3:**
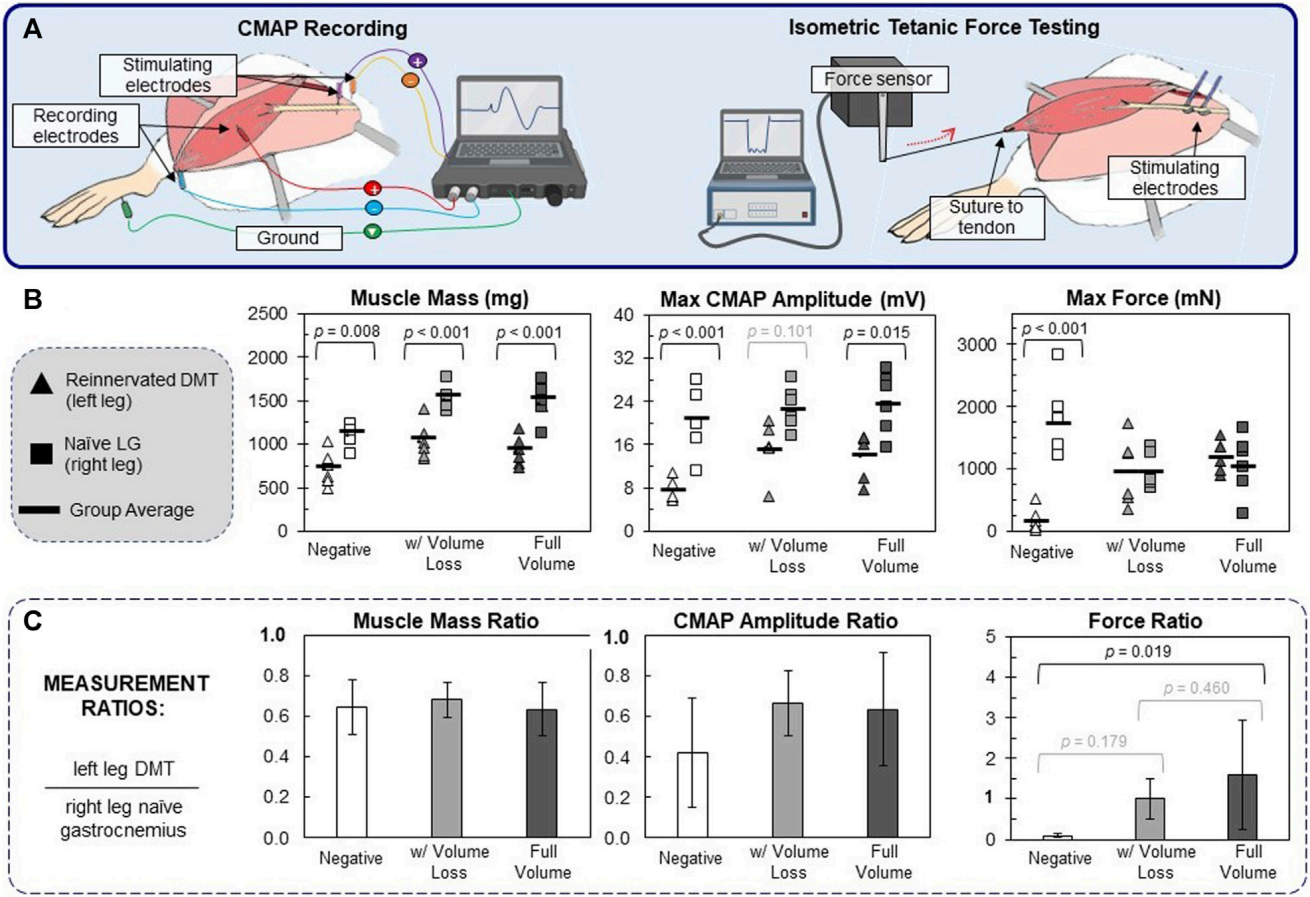
Functional outcomes of muscle reinnervation. **(A)** After reinnervation, CMAPs were recorded using needle electrodes and a clinical EMG recording system. ITFT was performed by suturing the distal end of the DMT to the lever arm of a force sensor. The sciatic nerve was stimulated upstream to generate the muscle force. **(B)** The raw values for each animal (*n* = 6 per group) are reported, in which triangles represent the left DMT measurement, squares represent the right naïve LG, and bold lines represent the muscle group average. One-way ANOVA (*df* = 5) for muscle mass (*F* = 18.2), maximum CMAP amplitude (*F* = 10.4), and maximum force production (*F* = 8.3) resulted in statistical differences (*p* < 0.001 for all ANOVA tests). Within all three surgical groups, DMTs had significantly lower mass than naïve LG muscles. For CMAP amplitudes, there was a general trend that DMTs produced lower values than naïve muscles. As for the results of ITFT, only the negative group (7 days of denervation) had significantly lower force production in DMTs than the naïve LG muscles of the same group. **(C)** For each individual animal, measurement ratios were calculated, with left leg DMT values being normalized by the same animal’s right leg naïve LG values. The averaged ratio values are reported for animals in each surgical group (*n* = 6 per group), plus/minus one standard deviation. One-way ANOVA (*df* = 2) showed only statistical differences in the maximum force ratios (*F* = 4.88 and *p* = 0.023). Both MR surgical groups had force ratios close to 1, but only MR in the full-volume group was significantly larger than that in the MR-negative group (*p* = 0.019).

CMAPs were recorded using a Sierra Summit clinical CMAP recording system (Cadwell Industries, Kennewick, WA, United States). Two-needle electrodes were placed on the sciatic nerve close to the hip to act as stimulating electrodes. Two-needle electrodes were also placed in the exposed DMT muscle or naïve gastrocnemius muscle (during left and right leg recordings, respectively). The positive electrode was placed in the center of the muscle belly, and the negative electrode was placed on the most distal part of the muscle near the Achilles tendon (Figure 3A). The ground electrode was inserted into the ipsilateral footpad. More detailed images of the recording setup can be found in Supplementary Figure S2A. To generate CMAPs, a constant current pulse was generated by the stimulating electrodes (anodic square pulse, duration 100 µs), starting at 0.5 mA and increasing in 0.5 mA increments until the CMAP reached a maximum baseline-to-peak amplitude. Across animal groups, 75% of all DMT muscles and 75% of all naïve gastrocnemius muscles were maximally stimulated at 2.5 mA. The CMAP’s onset time (ms) and maximum amplitude (mA) were recorded for later analysis (Supplementary Figure S3A).

### 2.3 Isometric tetanic force testing

After CMAP recordings, isometric tetanic force testing (ITFT) was performed on each muscle with the 300C Dual-Mode Lever Arm System and its accompanying biphasic current stimulator (Aurora Scientific, Ontario, Canada). The sciatic nerve was elevated onto a bipolar hook electrode, and the DMT, or gastrocnemius muscle tissue, being measured was lifted. The muscle’s distal insertion point near the Achilles tendon was transected and then sutured directly to the end of the force lever with a 4–0 nylon suture (Figure 3A; more detailed images of the recording setup can be found in Supplementary Figure S2B). The limb was immobilized at the knee and ankle using rubber bands secured to the surgical board, and the angle of the board was adjusted such that the muscle’s axis of contraction was orthogonal to the arm of the force lever.

For ITFT, the muscle stimulation had to be optimized for both stimulus current and muscle length. To find the optimal stimulation current, the muscle was stretched to be taught (but not overstressed) and then stimulated with 10 pulses at 10 Hz (biphasic, 100 µs) at 0.5 mA. The current was increased until the maximum twitch force of the muscle was achieved. More than 85% of muscle samples tested were maximally stimulated at either 1 mA or 1.5 mA. Next, the muscle must be stretched to its optimal length for maximum tetany, which was determined by stimulating the nerve with 10 pulses at 40 Hz (biphasic, 100 µs) and then sliding the lever arm slightly away from the muscle, repeating this until the maximum tetanic force measurement was found (Supplementary Figure S3B). After optimizing the muscle sample’s stimulation current and length, an automated program stimulated the nerve with 10 pulses at increasing frequencies from 1 Hz to 150 Hz, allowing the muscle to rest for 60 s between stimulation bursts. After the stimulation series was complete, the program would identify and record the maximum force (mN) produced for each stimulation frequency. Across all animal groups, 74.5% of samples (*n* = 47 total) were maximally stimulated with 80–150 Hz pulse delivery, and no sample was maximally stimulated below 50 Hz (Supplementary Figure S4).

### 2.4 Tissue histology

After the completion of ITFT, the muscle tissue was removed by transecting the origin near the knee, and its wet weight was recorded. The muscle was then bisected at the center, with half of the tissue being transferred into a 4% paraformaldehyde solution for 12 h, followed by storage in sterile PBS at 4°C. The other half was placed in a cryomold with OCT, which was immediately frozen using a cryo-block in a liquid nitrogen bath. The fixed tissue was embedded in paraffin, sectioned, and then stained with Masson’s trichrome, a histological stain that turns muscle cells a pinkish red, connective tissue blue, and cell nuclei black. The unfixed frozen tissue was sectioned using a cryotome (10 µm sections) and then fluorescently labeled using the same immunohistochemistry methods described by Bergmeister et al. (2017). The one difference in our methods was the secondary antibodies used; for our sections, tissue was labeled such that Type 1 fibers are blue (DAPI channel, 441 nm emission), Type 2a fibers are green (GFP channel, 520 nm emission), and Type 2b fibers are red (DSRed channel, 620 nm emission). Our full list of antibodies and dilutions can be found in Supplementary Table S1.

The stained cross sections were imaged using a Nikon ECLIPSE Ti microscope and digitally scanned using NIS-Elements software with a Hamamatsu ORCA Flash 4.0 monochromatic digital camera. TIFF files were analyzed using ilastik segmentation software. Two images were manually labeled to identify Type 1, Type 2a, and Type 2b fibers in the fluorescent samples. Then, an algorithm was trained on those labels to classify the image pixels of all the muscle samples (ilastik open-source software). The same segmentation process was used to identify muscle cells and connective tissue labeled in the Masson’s trichrome-stained sections. Individual muscle fibers were defined as masked particles with an area of 10^2^–10^4^ μm (Hsu and Cohen, 2013). FIJI open-source software was used to measure the area of the segmented images. The mean fiber area of all the discrete muscle fibers within a sample was calculated, and then these areas were summed to calculate the total muscle fiber area (mm^2^).

### 2.5 Data analysis and statistics

For analyses reported herein, each subject in the three surgical groups (negative, MR with volume loss, and MR full volume) was measured in both left and right hindlimbs, so data points were identified as being in 1 of 6 possible groups (36 total samples measured). One-way ANOVA (*df* = 5, n = 6 per group) was performed on raw values, and then inter-group significance was calculated using Tukey–Kramer HSD *post hoc* tests. In addition to comparing raw values from each limb of each animal, we recognize that all positive control limbs (right leg naïve LG muscles) are paired with a specific left leg DMT measurement, so some data are reported after first being normalized. That is, for each animal, the ratios of the left leg measurements to the right leg measurements were calculated. Not only does this normalize the DMT values, but it also reduces the number of groups for comparison. One-way ANOVA calculations were performed on the ratio values (*df* = 2, *n* = 6 per group) along with Tukey–Kramer HSD *post hoc* tests. Finally, multiple continuous variables that were measured (e.g., a muscle’s mass and its maximum CMAP amplitude) were also analyzed for linear correlation. Basic linear regression was performed to find the line of best fit, and Pearson correlation coefficients were calculated between the two continuous variables.

## 3 Results

### 3.1 DMTs have smaller muscle mass and CMAP amplitude but similar force output as naïve LG muscles

On the day of surgery, all rats had similar body mass (273 g ± 11 g) with no significant difference between any groups (Supplementary Figure S5A). The MR rats all had similar body mass at the time of takedown 100 days later and, on average, had grown 51.8% in mass from their date of surgery. In contrast, MR-negative animals measured at 7 days of denervation were only 4.95% (±0.67%) larger in mass, which is to be expected given the normal growth rate of young male rats. All of the DMT samples had significantly lower mass than positive controls, but none of the three surgical groups had average DMT masses that were significantly different from one another (Figure 3B). While unintuitive, this was not entirely unexpected for our team. Prior to these experiments, we performed CMAP collection in the same hindlimb model of MR with a cohort of animals that had an additional intermediate size, in which either 600 mg or 300 mg of the DMT tissue was removed at the time of MR surgery. We found that the post-reinnervation mass of the DMT muscles at 100 days did not significantly differ between the three differently sized groups (Supplementary Figure S6).

Similar to muscle mass, there is a trend in maximum CMAP amplitudes showing that DMTs have smaller amplitudes on average than positive controls (*F* = 10.41, *p* < 0.001) (Figure 3B). Despite reduced mass and CMAP amplitude in DMT samples, the raw ITFT measurements at 100 days were roughly the same for all groups (Figure 3B). The only group with a difference in the force output between left and right leg measurements was the MR-negative group after 7 days of denervation. When left leg measurements are normalized by their paired right leg measurements, a similar pattern emerges (Figure 3C). The average ratios for muscle mass and CMAP amplitude are all less than 1, with no statistical differences observed between groups: *df* = 2 and *F* = 0.25 for muscle mass and *F* = 1.79 for CMAP amplitude (*p* < 0.001 for both ANOVAs). There was some difference seen in the ratios of force output (*F* = 4.88 and *p* = 0.023) because of the dramatic difference between our MR-negative (7 days) group and the MR surgical groups. On average, DMT force output as a percentage of its paired naïve LG was greater than 100% in both the MR surgical groups, while the MR-negative DMTs produced only 9.6% (±6.3%). In many studies, it is standard to normalize force measurements (N) by the muscle’s mass (g), but doing so in this case makes the DMT force output significantly larger than the naïve LG muscles because of the group differences in mass. This topic is revisited in the Discussion section below.

### 3.2 DMTs display smaller muscle fiber areas but similar fiber types

**FIGURE 4:**
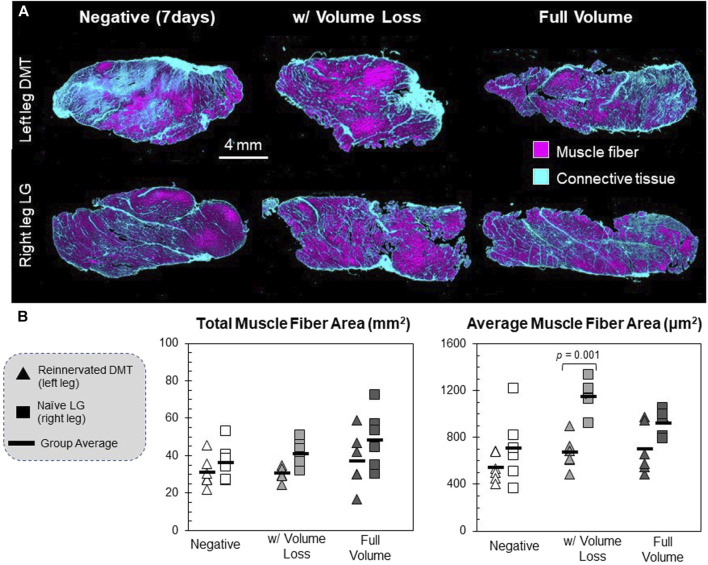
Histology of reinnervated muscle fibers. **(A)** Muscle tissue cross-sections were stained with Masson’s trichrome, which differentially stains muscle fibers and connective tissues. Scanned tissue images were classified by pixel using ilastik segmentation software. **(B)** Individual muscle fibers were identified and measured using FIJI open-source software. These are the raw values for each animal’s left and right limbs (*n* = 6 per group). The muscle fiber area was defined as any continuous area labeled “muscle fiber” that falls within the size range of 10^2^–10^4^ μm (Hsu and Cohen, 2013). The total muscle fiber area is the summed area of all the muscle fibers detected in a sample; *df* = 5, *F* = 4.42, and *p* = 0.004. The average muscle fiber area is the mean of all the discrete muscle fibers in a sample; *df* = 5, *F* = 8.73, and *p* < 0.001.

Cross sections of every muscle were imaged after being stained with Masson’s trichrome, a histological stain that differentiates between muscle cells and connective tissue (Figure 4A). Images were measured for the area of muscle fibers as a whole and individually, as well as the connective tissue area. There were no major differences between groups’ total cross-sectional area or connective tissue fraction (*F* = 2.28; *p* = 0.072 and *F* = 2.87; *p* = 0.031, respectively; data not shown). No DMT group was statistically different from positive controls when measured for *total* muscle fiber area, with only a trend toward smaller measurements (*F* = 4.42 and *p* = 0.004), but the reinnervated DMTs with volume loss had significantly lower average muscle fiber cross-sections than the positive control group (*p* = 0.001) (Figure 4B).

**FIGURE 5:**
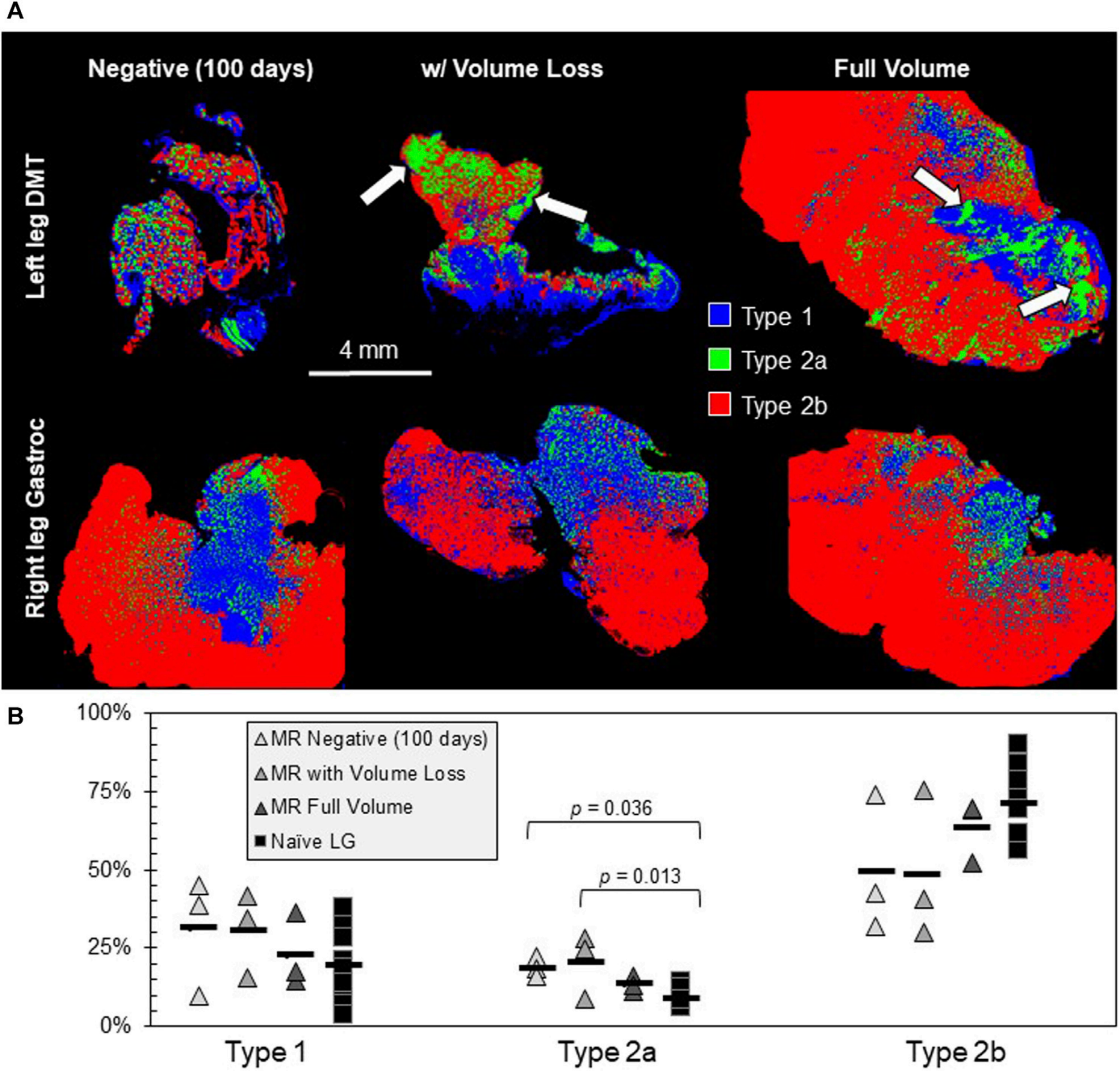
Muscle fiber type analysis. **(A)** Muscle tissue cross-sections were fluorescently labeled for Type 1, Type 2a, and Type 2b muscle fibers (*n* = 3 samples in the MR group and *n* = 9 samples in the Naïve LG group). The MR-negative DMTs being analyzed for fiber type were collected 100 days post-denervation. One hallmark of the reinnervated tissue that is present is an increased clumping of fiber types, especially Type 2a (white arrows). **(B)** The only significant difference seen between fiber type groups is a slight increase in Type 2a fibers for the MR-negative (100 days) and the MR with volume loss groups; *df* = 3, *F* = 6.05, and *p* = 0.007.

Different cross sections of each muscle were fluorescently labeled via immunohistochemistry, targeting the different myosin heavy chain proteins for Type 1, Type 2a, and Type 2b muscle fibers (Figure 5A). At 100 days (including for the MR-negative group in this case), the fractional distribution of fiber types was not extremely different between the three types of DMTs (*n* = 3 per group) and positive controls (*n* = 9), except for a slight increase in the percentage of Type 2a fibers for both DMTs in the MR-negative (100 days) group and the MR with the volume loss group (*df* = 3, *F* = 6.05, and *p* = 0.007) (Figure 5B). On visual inspection of the DMT slides, there is no notable pattern to distinguish groups, but the naïve LG muscles appear somewhat stereotyped. The rat LG has two distinct muscle heads, which are present in all naïve sections. The composition is mostly Type 2b fibers, and a mosaic of Type 1 and Type 2a fibers makes up the tissue near where the two heads meet. The only feature that is similar among all the DMT sections is a distinct clumping of fibers in this central region, especially of Type 2a fibers.

### 3.3 Variable tissue features influence muscle behavior irrespective of reinnervation

**FIGURE 6:**
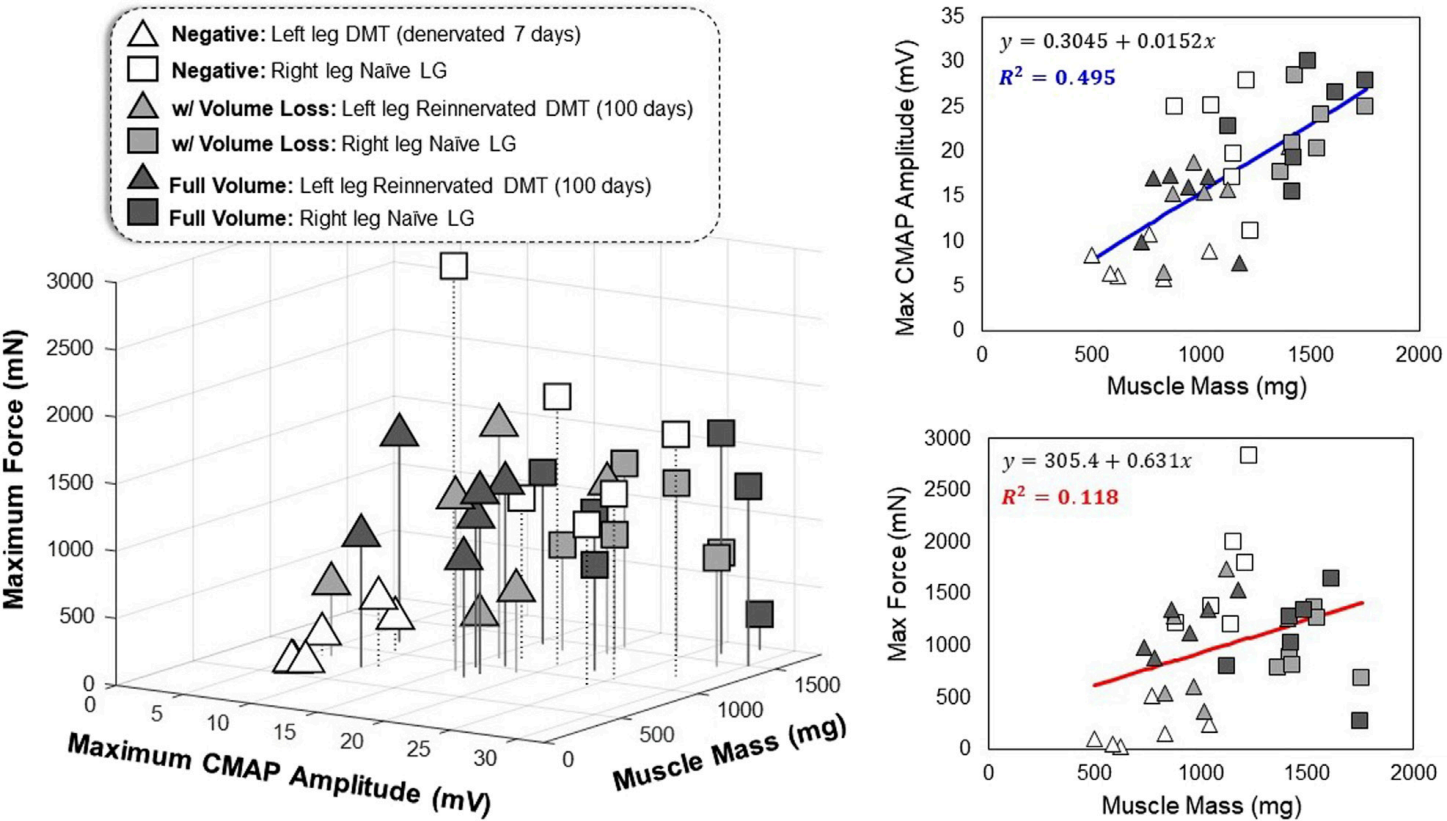
Multivariate relationships in functional outcomes All data points represent one leg of a single animal subject that was measured, where triangles represent the left leg DMT and squares represent the right leg naïve LG muscle (36 total data points). The axes for the 3D plot are muscle mass (mg) *versus* maximum CMAP amplitude (mV) *versus* maximum tetanic force production (mN). Linear regression was performed on the data (regardless of the reinnervation status) to produce best-fit equations as a function of muscle mass. The correlation observed between muscle mass and CMAP amplitude is stronger than the relationship seen between muscle mass and maximum force production (*R* ^*2*^ = 0.495 and *R* ^*2*^ = 0.118, respectively).

Generally, reinnervated muscles had lower mass and lower CMAP amplitudes than the positive controls, but it is possible that these two variables are related irrespective of the surgical group. The results of one-way ANOVA tests suggest that there is a significant difference between the groups that is not explained by chance alone (Figures 3–5); however, if we assume no distinction between the groups, can variation in muscle mass alone act as a predictor of functional outcomes? This hypothesis is supported by the results of linear regression, which show a weakly positive trend for CMAP amplitude with respect to muscle mass (Figure 6). This relationship, while broad, is significant based on a Pearson’s correlation test (*R* ^*2*^ = 0.495 and *p* < 0.001). Mass and force output have no obvious correlation (*R* ^*2*^ = 0.118 and *p* = 0.040), and therefore, something inherent to the MR surgical procedure might be affecting the measured outcomes of ITFT (Figure 6). Our expanded dataset shows a similar positive correlation between CMAP amplitude and average muscle fiber area (Supplementary Figures S5B, C), and the connective tissue fraction of the cross-sections showed an equally strong correlation with ITFT results, as compared to muscle mass (Supplementary Figures S5D, E).

## 4 Discussion

Regarding the effects of *vascularized DMT tissue size* on the outcomes of reinnervation, the evidence we have collected suggests that tissue mass and CMAP amplitude are positively correlated in rats after 100 days of reinnervation (Figure 6). Both the muscle mass and CMAP amplitudes of reinnervated DMTs are lower than naïve positive control muscles, but in this model, removing tissue during MR surgery did not significantly impact muscle mass 100 days later (Figure 3B). Our results also suggest that the muscle fiber cross-sectional area could be positively correlated with CMAP amplitude (*R* ^*2*^ = 0.208 and *p* = 0.001) (Supplementary Figure S5C), which is supported by other studies in rat hindlimb models of peripheral nerve injury; when transecting a nerve and coaptating it back together, leg circumference is an often-used indicator of the progress in nerve regeneration (Vennemeyer et al., 2015; Tatu et al., 2023). Our results are also similar to those studies in that very high intra-group and inter-animal variability among all the measurements prevents us from drawing strong statistical conclusions with the variables that we have analyzed (Schmalbruch, 1986; Tatu et al., 2023). With regards to muscle force production after reinnervation, our results do not demonstrate any clear patterns. For DMT muscles in both MR surgical groups, with and without volume loss, ITFT produced statistically similar results as compared to the contralateral naïve LG muscles at 100 days post-surgery, despite the DMT groups having significantly lower muscle mass (Figure 3B).

To better interpret our results, we should evaluate the force produced by our positive controls. For the MR surgical groups, the naïve LG muscles produced approximately 1 N of force, with a large standard deviation for both groups (Supplementary Figure S5A). This is lower than force measurements recorded from naïve medial gastrocnemius muscles by other groups who have used similar methods (but with younger animals and with different anesthesia protocols). MacIntosh and MacNaughton (2005) showed peak forces of approximately 4 N in 210 g rats while measuring maximal *twitch* force (50 µs stimulation pulses), and Jaspers et al. (1999) measured peak tetanic force of approximately 9 N in 350 g rats when stimulating at 1 kHz (100 µs pulses). One key difference between our methods and theirs is the muscle’s angle relative to the rest of the limb’s anatomy during testing. Both aforementioned studies aimed to replicate the ideal physiological conditions for the muscles tested. In another study, which more closely mimics our surgical, anesthesia, and ITFT methods performed by Isaacs et al. (2013), the naïve gastrocnemius muscle (medial and lateral heads together) produced on average 1.2 N of contractile force with slightly higher muscle mass on average than our positive controls. However, these force measurements were taken during muscle twitches (2 ms pulses) at supramaximal stimulation voltages. Additionally, we observed that tetanic force measured in naïve muscles at 7 days post-surgery was higher (1.8 N) than at 100 days post-surgery, implying that some other factor was reducing naïve LG muscle function in the surgical group rats. This could be due to factors such as the older age of the animals or some kind of bilateral reduction in muscle activity and strength as a secondary result of the MR surgery (De Haan et al., 1993; Powers and Jackson, 2008).

Next is the relationship between DMTs and naïve muscle forces: raw measurement values from DMTs produced similar values (−1 N) compared to positive controls. One logical way to normalize the raw force produced is with the muscle’s mass. However, this does not seem statistically appropriate in our case, as the surgical intervention significantly reduced the muscle mass. Normalization in this way would then indicate that the reinnervated DMTs produce significantly more force per mg of tissue than naïve LG muscles, and our experiments were simply not optimized for enough variables to draw any conclusions there. Our study of the effects of muscle size on reinnervation is limited in that it measures wet muscle mass and cross-sectional area but not muscle *volume* in mm^3^ as a metric of size. Muscle volume could be measured with 3D imaging techniques, either *in vivo* or *ex vivo*. Improving our 3D imaging methods would allow us to characterize the muscle boundary measurements with better details, as they might be more strongly related to muscle force output (Russell et al., 2000; Fukunaga et al., 2001).

Previous studies in the rat gastrocnemius muscle have shown that larger volumetric loss leads to reduced force output after 28–42 days of injury, but those muscles did not have the added insult of acute denervation, which reduces function and increases muscle atrophy (Merritt et al., 2010a; Merritt et al., 2010b). Conversely, few studies that look at recovery from denervation are performed in models that include volumetric muscle loss (Gilbert-Honick and Grayson, 2020). To evaluate how the biological processes of recovery from denervation *and* recovery from volume loss might impact one another, future work using our model should include control groups that only experience volume loss without any denervation. If we were to normalize our force measurements by the muscle mass, reinnervated DMTs would have a larger force per mg than positive controls because, on average, they had lower mass. Better control groups are required to determine if this is due to chance or if there is a consistent difference between volume–force relationships in the reinnervated muscle. One of the major weaknesses of our MR model in the LG muscle is the lack of strong evidence to identify the exact source of motor innervation for each muscle. We discuss this more below, but additional data with different muscle–nerve combinations are required to determine if changes in force per gram of tissue could become an indicator of reinnervation status in future studies.

None of our vascularized DMTs showed significantly more fibrosis or necrosis than the others (Figure 4), nor did one group show significantly different tetanic force at 100 days of reinnervation (Figure 3B). One of our unfortunate results is that at 100 days of reinnervation, the MR-*negative* group produced 98% (±52%) of the force output of paired positive controls (*n* = 5, data not shown) (Supplementary Figure S5). This recovery, despite our efforts to remove as much of the lateral tibial nerve branch as was possible, is why we decided to record from our final MR-negative cohort at 7 days of denervation. The development of unintended reinnervation should not have been a surprise; it is supported by evidence that rats have double the peripheral nerve growth rate post-injury compared to primates and generally show a higher capacity for regeneration (Gutmann et al., 1942; Danielsen et al., 1986). This can be both a benefit and a detriment. Beneficially, a faster nerve growth rate means that rats can be used for faster, high-throughput studies of reinnervation. In addition to the challenge of translating the work to human studies, the difficulty in intentionally preventing reinnervation in a rat DMT makes it more complicated to establish truly negative controls.

Muscle fiber types were measured after reinnervation because the results can sometimes provide clues about the source of muscle innervation, which would also help us troubleshoot the “unintended reinnervation” in our MR model (Bergmeister et al., 2019). Unfortunately, rat hindlimb muscles have a high composition of Type 2 fibers, including those innervated by both the tibial and peroneal nerves (Armstrong and Phelps, 1984; De Feyter et al., 2006); so, fiber type composition was not a useful identifier for unique surgical groups. MR-negative (100 days) and MR with volume loss DMTs both showed a statistically larger fraction of Type 2a fibers and a slightly lower average percent of Type 2b fibers, but this could be a result of denervation on its own because there is evidence that Type 2b fibers do convert to Type 2a in denervated rat hindlimb muscles (Pette and Vrbová, 1985; Windisch et al., 1998; Kostrominova et al., 2005). For future studies, we must consider that the best way to confirm the source of innervation for a muscle is through electrical stimulation. In our CMAP collections, we stimulated the sciatic nerve very far upstream from the site of tibial nerve transection. We did this for the following two reasons: to keep the stimulator as far from the recording electrodes as possible for reducing stimulus artifact interference with the signal and to keep the stimulation parameters the same for all animals on both limbs, regardless of the target muscle’s source of innervation. Despite the benefits of this choice, it causes mechanical artifacts to appear from nearby naïve muscles that are also being stimulated during CMAP recording. The best, noise-free CMAP recording is one in which only the target muscle is being stimulated. In our future studies, we can attempt to achieve this either by moving the stim electrode closer to the target tissue or by denervating all of the off-target muscles.

The most important predictor of functional outcomes in a reinnervated DMT is the passage of time (Gordon, 2020). We collected data for the MR-negative group at 7 days because innervation was expected to be minimal, making it a better negative control. This proved to be a good choice, but it only emphasizes that the data collected at single time points do not tell the full story of recovery. Reinnervation is a time-dependent process, and therefore, chronically monitoring DMTs throughout their recovery with high temporal resolution will lead to a stronger understanding of how the different variables interact in this macro-scale process. Chronically implanted electrodes are no longer a technological barrier to these studies, but employing in large-scale experiments still remains challenging (Mastinu et al., 2018; Muceli et al., 2018). The ideal experimental design would instead collect as much data as possible from a DMT using minimally invasive methods (e.g., ultrasound imaging and grip strength tests) at as many time points as possible (Wang et al., 2008; McGillivray et al., 2022; Morag et al., 2022). Without the ability to chronically monitor the same muscle at different time points as it is reinnervated, the number of rats increases by *n* with each new time point added to any longitudinal study. Our future studies will focus on using implantable electrodes to chronically monitor various DMTs as they transition from fully denervated to fully reinnervated. Chronic monitoring also improves our control groups because normal muscle can be equally impacted functionally by the animal’s age, weight, and activity level. This improved longitudinal data will be acquired with the long-term goal of developing neuromuscular models. More accurate modeling of reinnervation and muscle remodeling could give future clinicians the predictive power necessary to estimate the electrophysiology or kinesiology of a DMT *after* reinnervation based on its highly unique anatomy at the time of MR surgery.

## Data Availability

The raw data supporting the conclusion of this article will be made available by the authors, without undue reservation.
