# Supplementary material for: Volume loss during muscle reinnervation surgery is correlated with reduced CMAP amplitude but not reduced force output in a rat hindlimb model
Source: Front Physiol. 2024 Feb 15;15:1328520. doi: 10.3389/fphys.2024.1328520 (PMC10902164; doi:10.3389/fphys.2024.1328520)
Supplement: Supplementary file 1 [file DataSheet1.PDF]

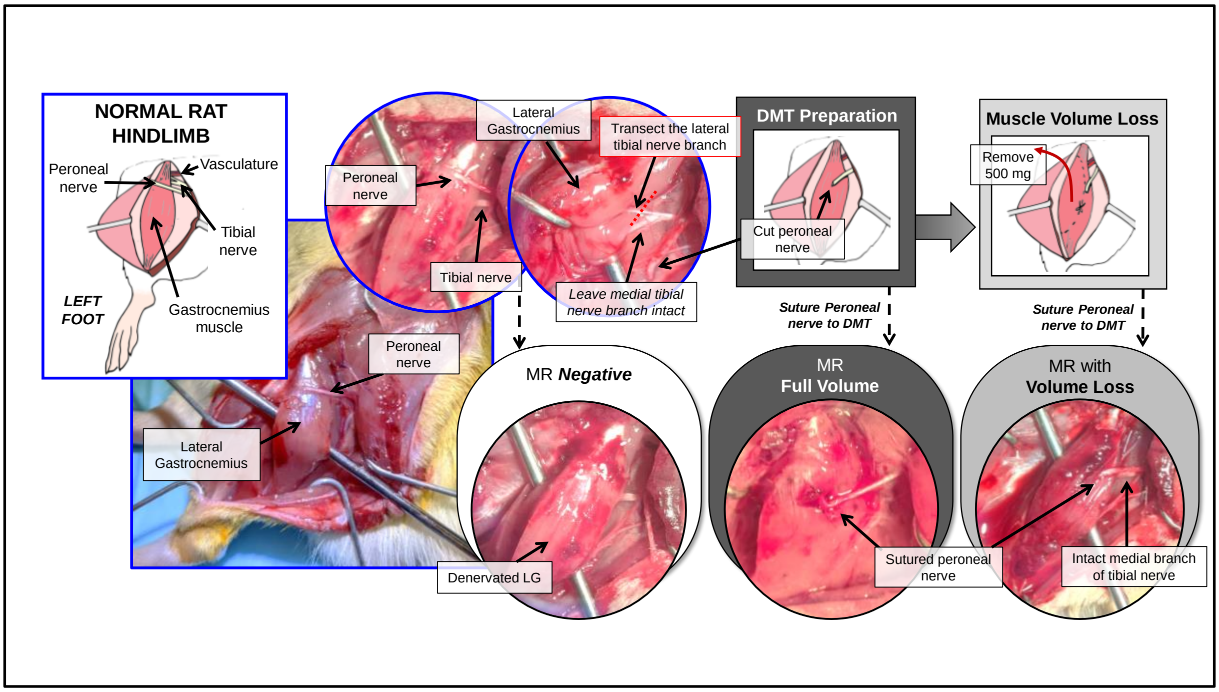

<u>Supplemental Figure S1:</u> Surgical variations of Muscle Reinnervation (MR) procedure in the rat hindlimb with and without volume loss.

In this rat hindlimb model of MR, the gastrocnemius muscle is used as the source for the denervated muscle target (DMT), and the peroneal nerve acts as the cut donor nerve. First the lateral gastrocnemius must be relieved of its endogenous innervation by cutting the lateral branch of the tibial nerve (while leaving the medial branch to the medial gastrocnemius intact). When the nerve branch is cut, the LG visibly twitches, and the DMT has been created. Reinnervation is performed by suturing the cut peroneal nerve to the LG DMT either with or without volume loss. To model volumetric muscle loss, 500 mg of muscle tissue was extracted in longitudinal strips before MR.

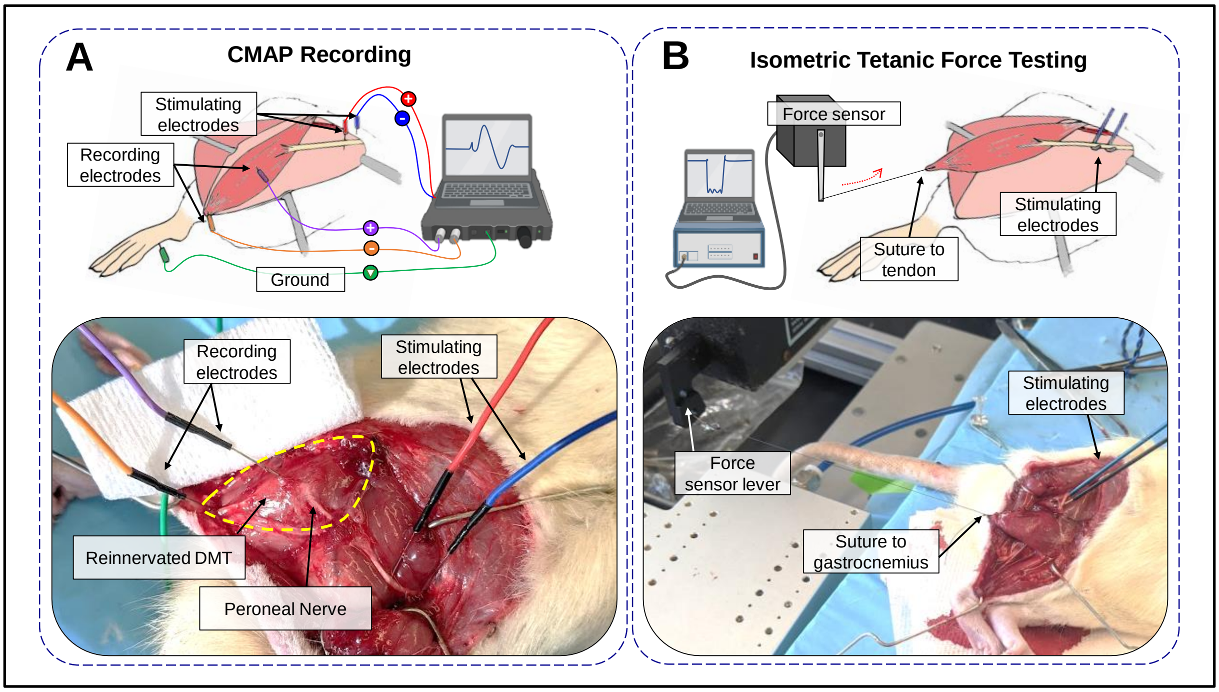

<u>Supplemental Figure S2:</u> Measuring muscle function after one hundred days of reinnervation. (A) Compound motor action potentials (CMAPs) were recorded using needle electrodes. The positive electrode was placed in the center of the muscle belly, and the negative was placed near the lateral Achilles tendon. The bipolar stimulating electrodes were placed on the sciatic nerve near the hip. The electrode placements were adjusted to maximize the CMAP amplitude recorded using a Sierra Summit clinical CMAP system. **(B)** Isometric tetanic force testing (ITFT) was performed by cutting the distal Achilles tendon of the reinnervated muscle, and then suturing the tendon to the end of a force sensor lever (Aurora Scientific). The muscle length was adjusted by rolling the lever on a level track with the muscle orthogonal to the lever arm. For every muscle measured, the optimal muscle length was found by measuring the force while stimulating the sciatic nerve upstream with bipolar hook electrodes. Afterwards, an automated program stimulated the nerve at increasing frequencies, and the maximum force produced was recorded.

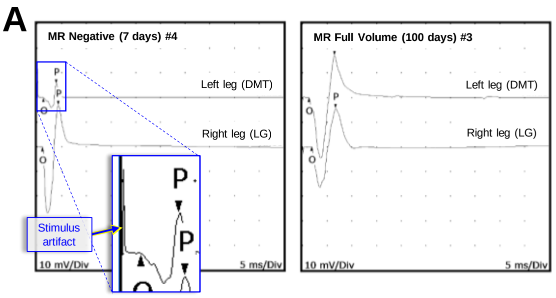

Animal	Leg	Onset (ms)	O-P Amp (mV)
MR Negative (7 days) #4	Left (DMT)	1.3	5.8
MR Negative (7 days) #4	Right (Naïve LG)	1.1	17.1
MR Full Volume (100 days) #3	Left (DMT)	1.3	17.2
MR Full Volume (100 days) #3	Right (Naïve LG)	1.9	15.5

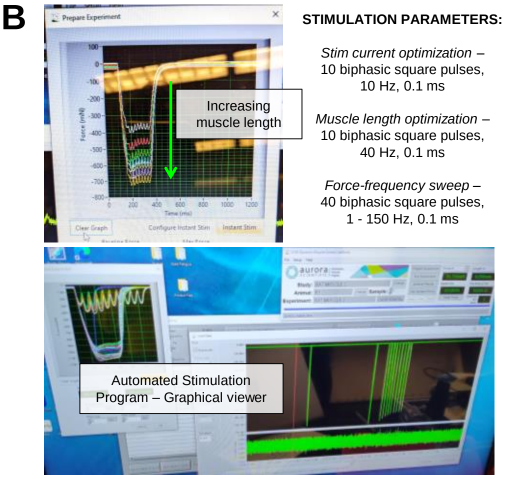

<u>Supplemental Figure S3:</u> Signal processing for CMAPs and ITFT.
(A) CMAPs were recorded with a clinical Sierra Summit system, so raw time-domain signals could not be exported. Shown here is the printout image from two separate animals, with both the left and right leg CMAPs overlaid on the same plot. An inlay shows that after the software initiates the nerve stimulus upstream (monophasic anodic square pulse, 0.1 ms). The stimulus current was increased until maximum CMAP amplitude was achieved, and that waveform was measured for its onset time (post stimulus) and its maximum amplitude. **(B)** ITFT was performed with an Aurora Scientific system. After the muscle is attached to the force lever, the optimal stimulus amplitude must be determined. This is done by stimulating the taught muscle with increasing current amplitudes until maximum twitch force is achieved (10 biphasic square pulses, 10 Hz, 0.1 ms). At that optimized stimulation current, the muscle is then adjusted for optimal length. Stimulated with 10 pulses at 40 Hz, the muscle is slowly stretched until maximum tetany is achieved. Finally, an automated program would stimulate the muscle at a range of frequencies (10 pulses at 1, 10, 20, 40, 50, 60, 80, 100, and 150 Hz). The maximum force produced at each frequency is measured and recorded.

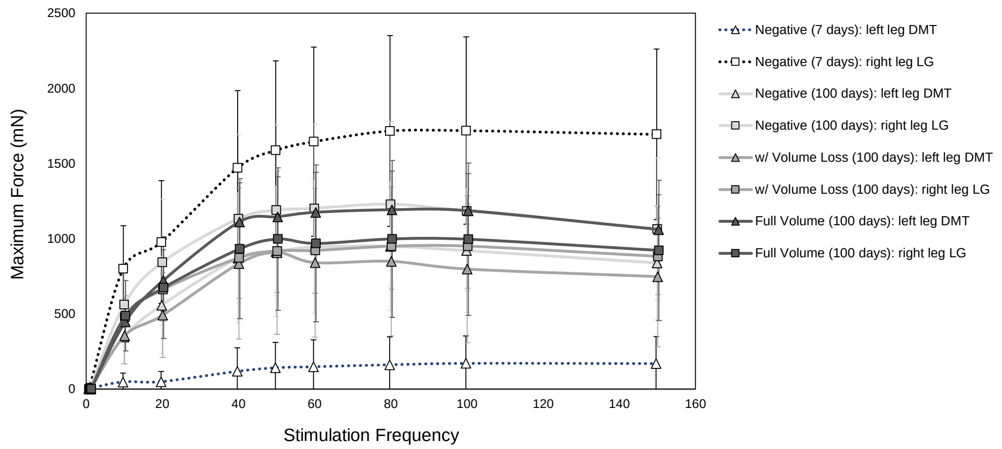

<u>Supplemental Figure S4:</u> Stimulation Frequency Sweep for ITFT

To acquire the maximal tetanic force from each muscle sample, it was stimulated at varying frequencies (biphasic 100 μs pulses) after optimizing for stimulation current and muscle length. Shown here are the group averages ± standard deviation for all of the surgical groups evaluated (n = 8 per group, except n = 7 for Negative 100 days right leg LG). Over 70% of samples were maximally stimulated between 80 Hz and 150 Hz, with no sample being maximally stimulated below 50 Hz.

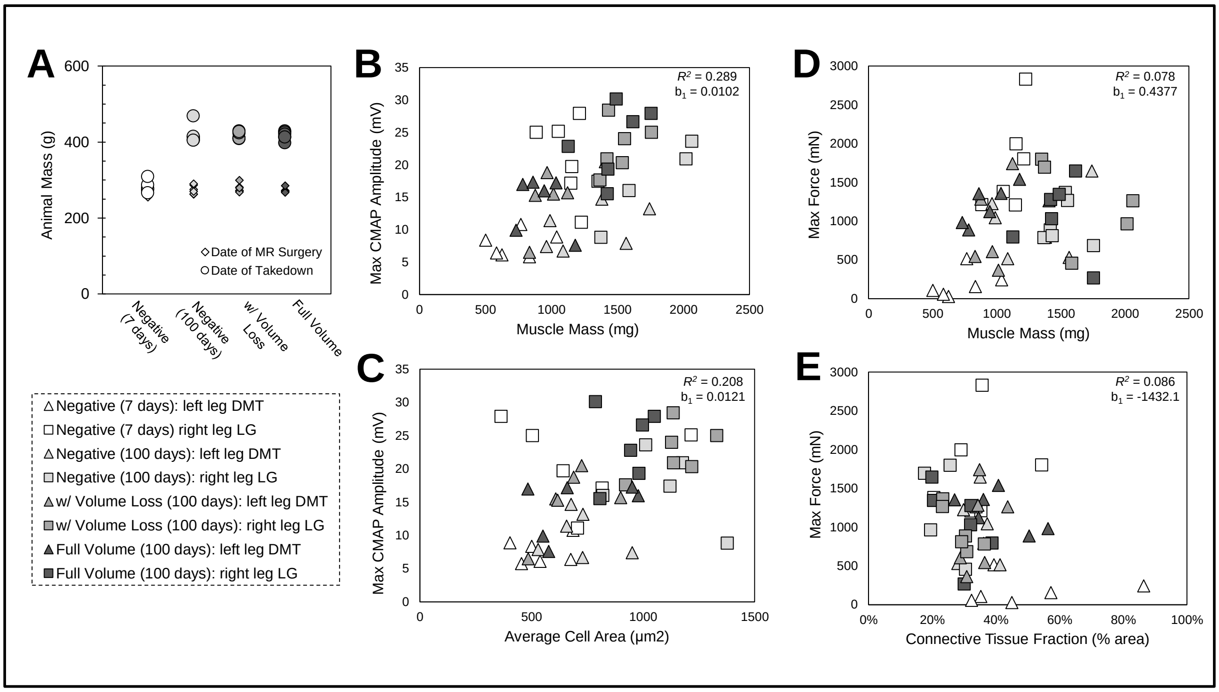

<u>Supplemental Figure S5:</u> Even more multivariate relationships.
(A) Animal mass for each group at the time of surgery and the time of sacrifice. Each point represents a single animal (n = 6 per group). **(B-D)** These data represent one leg of a single animal subject that was measured, where triangles represent the left leg DMT, and squares represent right leg gastrocnemius muscles (47 total data points). This dataset includes the MR Negative group that was recorded from after 100 days of denervation (n = 6). Shown here are the observations of: **(B)** muscle mass (mg) versus maximum CMAP amplitude (mV), **(C)** average cross-sectional area of muscle fibers (µm^2) versus maximum CMAP amplitude, **(D)** muscle mass versus maximum tetanic force production (mN), and **(E)** connective tissue fraction (as a percentage of total cross-sectional area) versus maximum tetanic force production (mN). Reported for each relationship is the R^2 value from Pearson correlation test and the slope of best fit from linear regression (b_1).

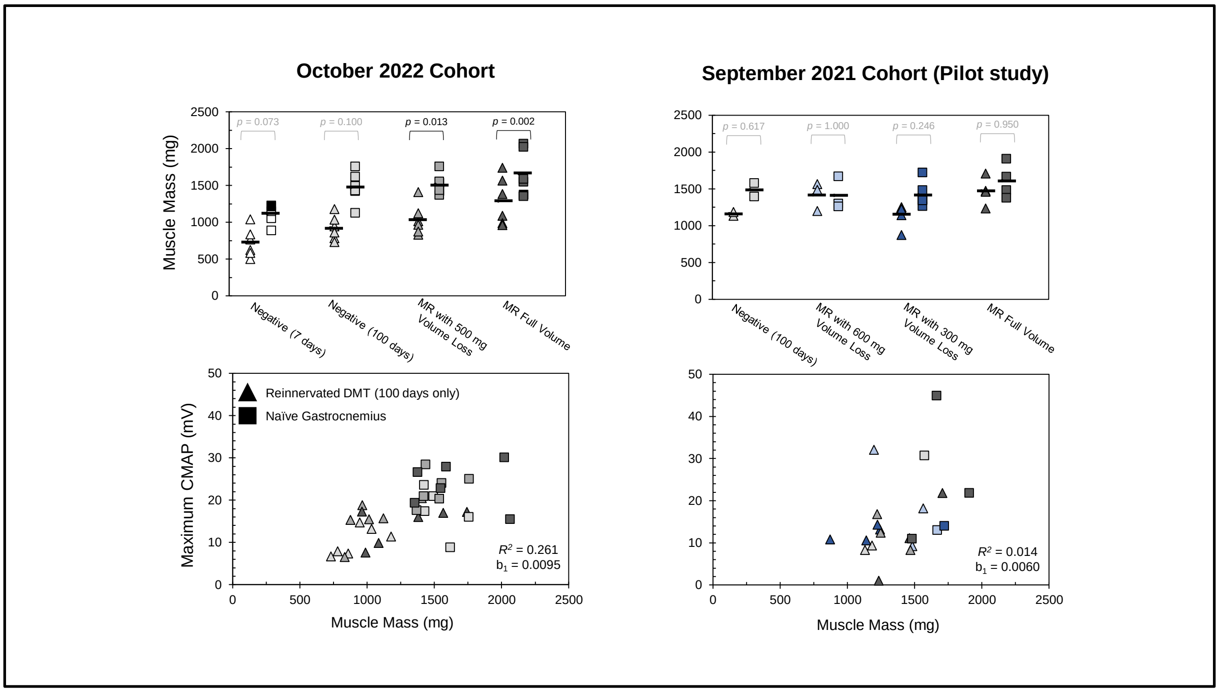

<u>Supplemental Figure S6:</u> Mass and CMAP Amplitude in two different cohorts.
The data presented in this manuscript were recorded in October 2022. Previously, we had collected CMAP data from a cohort that received the same surgery; these data were collected in September 2021. CMAPs were collected the same way in both cases: the left leg DMT was recorded first (triangles), and the right leg naïve gastrocnemius was measured second (squares). In addition to MR Negative (n = 2) and MR Full Volume (n = 4) groups, the 2021 cohort had 2 versions of MR with Volume Loss: -600 mg (n = 3) and -300 mg (n = 6). No statistical differences were found after 100 days of reinnervation between the different groups in terms of muscle mass. A similar linear trend was seen between muscle mass and maximum CMAP amplitude. These scatter plots show only data points collected at 100 days post-surgery (35 total data points for 2022 cohort, 21 data points for the 2021 cohort).

Fiber Type	Primary Antibody	Primary Ab Dilution	Secondary Antibody	Secondary Ab Dilution
Type 1	**BA-F8** (AB_10572253)	1:50	**Alexa Fluor 350 IgG2b** (AB_2535777)	1:250
Type 2a	**SC-71** (AB_2147165)	1:600	**Alexa Fluor 488 IgG1** (AB_2535764)	1:250
Type 2b	**BF-F3** (AB_2266724)	1:100	**Alexa Fluor 594 IgM** (AB_2535713)	1:250

Supplemental Table S1: Antibody dilutions for immunohistochemistry of muscle tissue cross-sections.
This table outlines the names and dilutions of the primary and secondary antibodies used for immunohistochemistry of muscle tissue slices. The primary antibodies were sourced from Developmental Studies Hybridoma Bank, and secondaries were acquired from Molecular Probes.
